# An improved blood hemorrhaging treatment using diatoms frustules, by alternating Ca and light levels in cultures

**DOI:** 10.1007/s42995-023-00180-3

**Published:** 2023-08-18

**Authors:** Qinfeng Li, Zheng He, Hussein. E. Rozan, Chao Feng, Xiaojie Cheng, Xiguang Chen

**Affiliations:** 1 College of Marine Life Science, Sanya Oceanographic Institution, Ocean University of China, Qingdao, 266003 China; 2 Laoshan Laboratory, Qingdao, 266237 China; 3 Department of Biochemistry, Faculty of Agriculture, Al-Azhar University, Cairo, Egypt

**Keywords:** Hemorrhage control, Diatom biosilica, Ca^2+^ biomineralization, Hemolysis rate, Hemostatic effect

## Abstract

Hemorrhage control requires hemostatic materials that are both effective and biocompatible. Among these, diatom biosilica (DBs) could significantly improve hemorrhage control, but it induces hemolysis (the hemolysis rate > 5%). Thus, the purpose of this study was to explore the influence of Ca^2+^ biomineralization on DBs for developing fast hemostatic materials with a low hemolysis rate. Here, CaCl_2_ was added to the diatom medium under high light (cool white, fluorescent lamps, 67.5 µmol m^−2^ s^−1^), producing Ca-DBs-3 with a particle size of 40–50 μm and a Ca^2+^ content of Ca-DBs-3 obtained from the higher concentration CaCl_2_ group (6.7 mmol L^−1^) of 0.16%. The liquid absorption capacity of Ca-DBs-3 was 30.43 ± 0.57 times its dry weight; the in vitro clotting time was comparable to QuikClot^®^ zeolite; the hemostatic time and blood loss using the rat tail amputation model were 36.40 ± 2.52 s and 0.39 ± 0.12 g, which were 40.72% and 19.50% of QuikClot^®^ zeolite, respectively. Ca-DBs-3 showed no apparent toxicity to L929 cells (cell viability > 80%) and was non-hemolysis (the hemolysis rate < 2%). This study prepared Ca-DBs-3 with a rapid hemostatic effect and good biocompatibility, providing a path to develop diatom biosilica hemostatic materials.

**Supplementary Information:**

The online version contains supplementary material available at 10.1007/s42995-023-00180-3.

## Introduction

Uncontrolled bleeding is a leading cause (nearly 40%) of death in military and civilian trauma (Eastridge et al. 2011; Sauaia et al. 1995; Teixeira et al. 2007). Rapid application of bleeding control can effectively prevent bleeding-related death (Kragh et al. 2009). Therefore, developing efficient hemostatic agents is critical. QuikClot^®^ (QC) is an inorganic zeolite that can powerfully treat fatal bleeding and save lives (Alam et al. 2005; Rhee et al. 2008). QC is considered to be one of the fastest and the least bleeding hemostatic agents in commercial products with many advantages, including stability, portability, and non-biological toxicity, and it has been used in the emergency treatment of bleeding in wars, traffic accidents, and other emergency situations (Li et al. 2013; Wright et al. 2004a). However, QC has an exothermic effect when used to treat bleeding, which will almost certainly result in secondary injury to the wound (Wright et al. 2004b). Therefore, safer fast hemostatic materials still need to be developed.

Diatom biosilica (DBs) has been found to stop bleeding rapidly and has no exothermic effect (Feng et al. 2016; Luo et al. 2021; Tramontano et al. 2020; Uthappa et al. 2018, 2019). Diatoms are unicellular eukaryotic algae found in almost all types of aquatic environment, including marine and freshwaters (Armbrust 2009; Falciatore and Bowler 2002) and are extremely diverse, with over 1 × 10^5^ known species (Mann and Droop 1996). Diatoms have an inorganic biosilica cell wall called a frustule, which is primarily composed of amorphous silica and distributed with numerous micro-nano pores (Dobrosielska et al. 2020; Hildebrand and Lerch 2015; Kröger and Brunner 2014; Zurzolo and Bowler 2001). A high porosity will result in a high absorption rate, which can rapidly agglutinate coagulation factors and accelerate the occurrence of coagulation cascade reactions (Li et al. 2012; Rhee et al. 2008).

Although DBs could rapidly cease bleeding, they have a hemolytic effect (the hemolysis rate > 5%) (Feng et al. 2016). Chitosan of marine origin is widely used in biomedical materials research (Deng et al. 2020). To decrease the hemolysis rate, chitosan was used to coat the surface of DBs and was found to be effective (Feng et al. 2016). However, chitosan will obstruct the porous structure of DBs, reducing their capacity for liquid absorption. Additionally, the chitosan-DBs mixture is an organic–inorganic composite material, and chitosan is sticky and will adhere to the wound, tearing the granulation tissue and causing allergies (Wang et al. 2021a, b). Recently, our research group added CaCl_2_ to the diatom culture medium and obtained an inorganic Ca-biosilica that was relatively pure (Li et al. 2018). Ca-biosilica’s hemolysis rate was less than 2%, implying that adding Ca^2+^ to DBs could reduce the hemolysis rate. However, the relationship between CaCl_2_ concentration and the amount of Ca^2+^ biomineralization is not well understood.

Application properties are based on the material's physical properties. The physical structure of post- harvested DBs can be changed by treating them with chemical reagents, such as SnO_2_ and hydrofluoric acid (Weatherspoon et al. 2007; Zhang et al. 2011). Chemical reagents can modify the physical properties of DBs. However, these procedures are complex, costly, and leave harmful residue. Thus, gentler modifications are needed. Since the frustule is prepared in the laboratory via the culture of living diatoms, the culture medium and culture conditions will affect the growth and biomineralization of diatoms, such as the frustules’ morphology and structure. Wei Li et al. found high light stimulated diatom’s growth and made diatom smaller (Li et al. 2023). Yanyan Su et al. investigated the effect of varying light intensities on the diameter of the frustule and discovered that as light intensity increased, the diameter of the frustule decreased (Su et al. 2015). Lulu Wang et al. described three types of frustules with a rapid hemostatic effect and the hemostatic effect increased with decreasing frustule size (Wang et al. 2019). 

To develop hemostatic materials that are effective and biocompatible, it is worthwhile to investigate the effects of light intensity and CaCl_2_ on the frustule microstructure. In this study, we investigated the effect of 4.05, 40.5, and 67.5 µmol m^−2^ s^−1^ (cool white, fluorescent lamps) on diatom growth and studied the impact of CaCl_2_ on diatoms under 67.5 µmol m^−2^ s^−1^. The microstructure, biomineralized Ca^2+^ content, liquid adsorption capacity, and hemostatic properties of the frustules were investigated to establish a relationship between the frustule’s physical and chemical properties and hemostatic efficacy.

## Results and discussion

### Effect of white light intensity on diatoms

The effect of light intensity on diatom’s carrying capacity was studied at three intensities (cool white, fluorescent lamps: 4.05, 40.5, 67.5 µmol m^−2^ s^−1^, Supplementary Fig. S1). Diatoms continued to multiply under the light of 4.05, 40.5, and 67.5 µmol m^−2^ s^−1^ during the 7-day incubation period, and the maximum carrying capacity of diatoms were reached at 6.98 × 10^5^, 8.07 × 10^5^ and 8.86 × 10^5^ cells L^−1^, respectively. The number of diatoms increased with increasing light intensity (*P* < 0.05), indicating that the increase in light intensity favored the cells division of diatoms in the range of 4.05–67.5 µmol m^−2^ s^−1^. The growth curves did not follow the typical sigmoidal curves due to the overly dense number of initial diatom inoculations. However, the results and findings are still useful. In future optimization experiments: (1) diatoms will be acclimated to the light conditions in an exponential phase acclimation culture for 10 generations; (2) the initial inoculum density of diatoms will be reduced to establish the typical sigmoidal growth curves; and (3) growth rate and carrying capacity will be calculated separately to guide industrial applications based on the exponential and steady-state growth periods of the diatoms.

SEM and TEM were used to examine the microstructure of DBs-4.05, DBs-40.5, and DBs-67.5. SEM analysis showed that DBs-4.05, DBs-40.5, and DBs-67.5 were cylindrical in shape, typical of the species, but had different dimensions (Supplementary Fig. S2A). The diameters of DBs-4.05, DBs-40.5, and DBs-67.5 were 90–100 μm, 50–60 μm, and 40–50 μm, respectively. TEM showed no significant difference in the pore size structure of DBs-4.05, DBs-40.5, and DBs-67.5 (Supplementary Fig. S2B). The tertiary pore structure was: 1–1.5 μm for the first-order aperture, 200–250 nm for the second-order aperture, and 50–100 nm for the third-order aperture. From 4.05 to 67.5 µmol m^−2^ s^−1^, white light affected the cell size but not the micropore size, with size decreasing as light intensity increased.

A BET analysis was conducted to better understand the effect of light intensity on the microstructure of DBs (Supplementary Fig. S3; Table S1). DBs-67.5 had the largest specific surface area (7.26 m^2^ g^−1^) and total pore volume of 0.016 cm^3^ g^−1^. As the intensity of the light increased from 4.05 to 67.5 µmol m^−2^ s^−1^, the specific surface area and total pore volume of frustules increased. The average pore diameter was around 8–10 nm. The Barrett-Joyner-Halenda (BJH) pore diameter was 2.39–2.41 nm. The average pore diameter and BJH pore diameter were not affected by the light intensity.

**Fig. 1:**
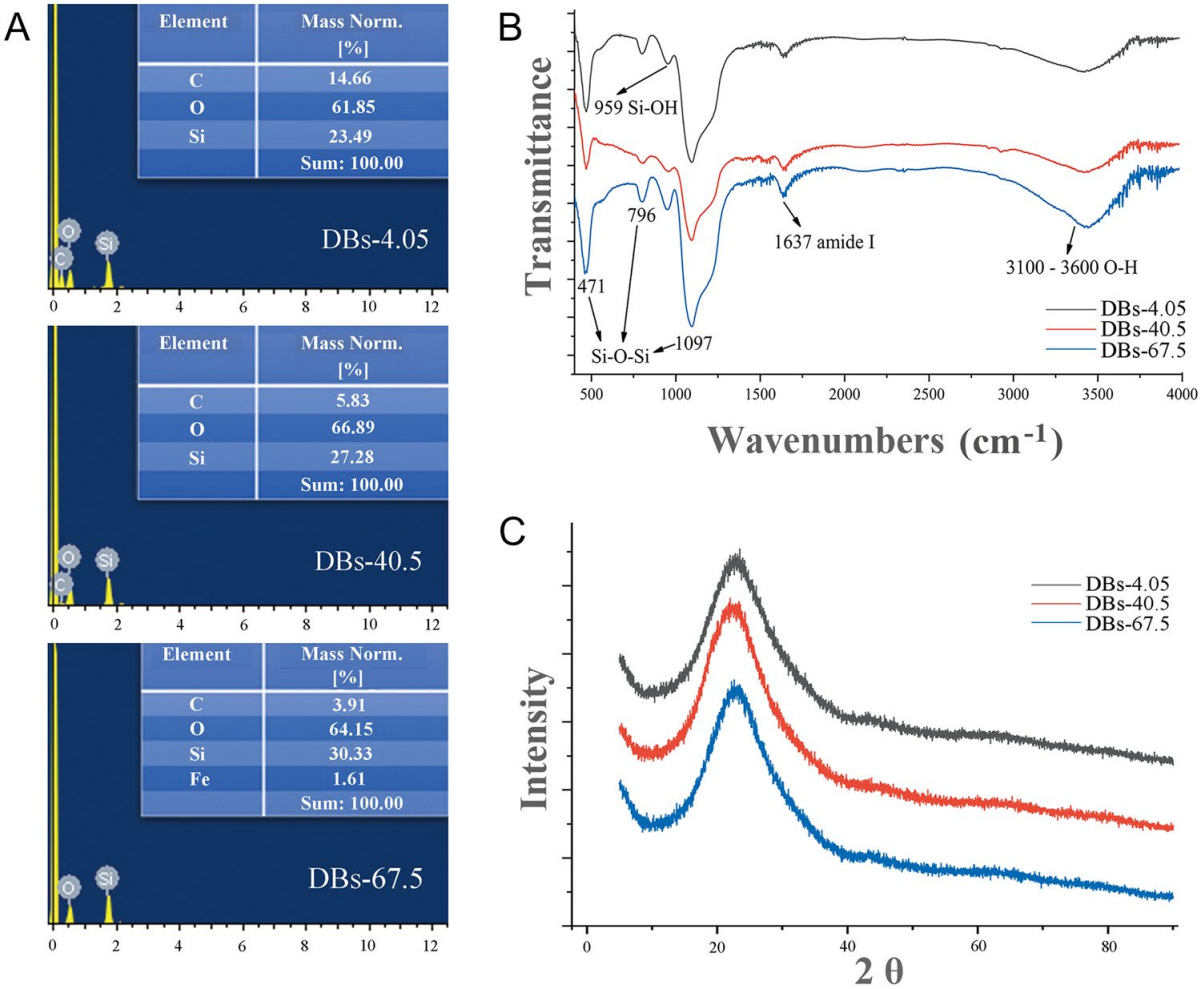
EDXS (**A**), FTIR (**B**), and XRD (**C**) spectra of DBs-4.05, DBs-40.5, and DBs-67.5

EDXS, FTIR, and XRD were used to determine the elemental composition, groups, and crystal form of DBs-4.05, DBs-40.5, and DBs-67.5, respectively. Elements C, O, and Si were detected in DBs-4.05, DBs-40.5, and DBs-67.5. However, only Fe was detected for DBs-67.5 (Fig. 1A). The higher light intensity may be responsible for the appearance of Fe. The FTIR curves of DBs-4.05, DBs-40.5, and DBs-67.5 were similar (Fig. 1B). The peaks at 471 cm^−1^, 796 cm^−1^, and 1097 cm^−1^ all correspond to Si–O-Si; the peak 959 cm^−1^ corresponds to Si–OH; the peak 1637 cm^−1^ corresponds to amide I (Zając et al. 2015); the peaks 3100–3600 cm^−1^ represent O–H. XRD analysis revealed that DBs-4.05, DBs-40.5, and DBs-67.5 were composed of amorphous silica (Fig. 1C). When exposed to white light at 4.05, 40.5, and 67.5 µmol m^−2^ s^−1^, DBs were similar in disk morphology, tertiary pores, surface clusters, and crystallization but differed in diatom growth rate, particle size, the specific surface area, total pore volume and ion biomineralization. An earlier study found that the smaller the frustule size, the more potent the hemostatic effect (Wang et al. 2019). Thus, 67.5 µmol m^−2^ s^−1^ white light was chosen to investigate the Ca^2+^ biomineralization on DBs because this wavelength showed the smallest frustule particle size, the largest specific surface area, the largest total pore volume, differences in ion biomineralization, and the fastest diatom growth rate.

### Effect of CaCl_2_ on diatoms

CaCl_2_ was added to the diatom medium at concentrations of 1.675, 3.35, and 6.7 mmol L^−1^ to determine its effect on diatom growth (Supplementary Fig. S4). Within 0–8 days, no significant difference in diatom number was observed between DBs (control), Ca-DBs-1, and Ca-DBs-2. Within 0–2 days, no significant difference was found in diatom number between DBs and Ca-DBs-3, but from day 2 to day 8, Ca-DBs-3 had significantly lower diatom number than DBs (*P* < 0.01).

At concentrations ranging from 1.675 to 3.350 mmol L^−1^, CaCl_2_ exhibited no detectable inhibitory effect on the diatom’s carrying capacity. However, at the higher concentration (6.7 mmol L^−1^), CaCl_2_ reduced the diatom’s carrying capacity. Frustule formation is an essential step in the division of diatom cells. The CaCl_2_ concentration as high as 6.7 mmol L^−1^ may affect the frustule biomineralization process and the diatoms’ cell division rate. The growth curve did not follow the typical sigmoidal curves due to the initial cultures were too dense. But these results and findings remain informative. In our future optimization experiment, diatoms will be acclimated to the Ca^2+^ conditions in an exponential phase acclimation culture for 10 generations. The initial cultures will be reduced to establish the typical sigmoidal growth curves. And a two-way ANOVA will be used to analyze the effects of light and Ca^2+^ on the growth rate and carrying capacity of diatoms for direct adaptation to industrial production.

SEM and TEM were used to examine the surface morphology and pore structure of DBs (control) and Ca-DBs (Ca-DBs-1, Ca-DBs-2, and Ca-DBs-3). Girdle bands connected the epitheca and the hypotheca of DBs and Ca-DBs (Falkowski et al. 1998; Field et al. 1998; Smetacek 1999). DBs and Ca-DBs were disc-shaped with a dense radial arrangement of graded circular/hexagonal and micro-nano pores (Supplementary Fig. S5). The average diameter of DBs and Ca-DBs was 40–50 μm; the maximum aperture was 1–1.5 μm; the second aperture was 200–250 nm; the minimum aperture was 50–100 nm. According to the BET analysis, the specific surface area of DBs and Ca-DBs was around 6–7 m^2^ g^−1^; the average pore diameter was 8–10 nm; the BJH pore diameter was around 2 nm; the total pore volume was around 0.015 cm^3^ g^−1^. DBs and Ca-DBs had similar specific surface areas and pore diameters (Supplementary Fig. S6; Table S2). The morphology and pore size of DBs and Ca-DBs are identical, indicating that CaCl_2_ did not affect the frustule’s shape or size.

**Fig. 2:**
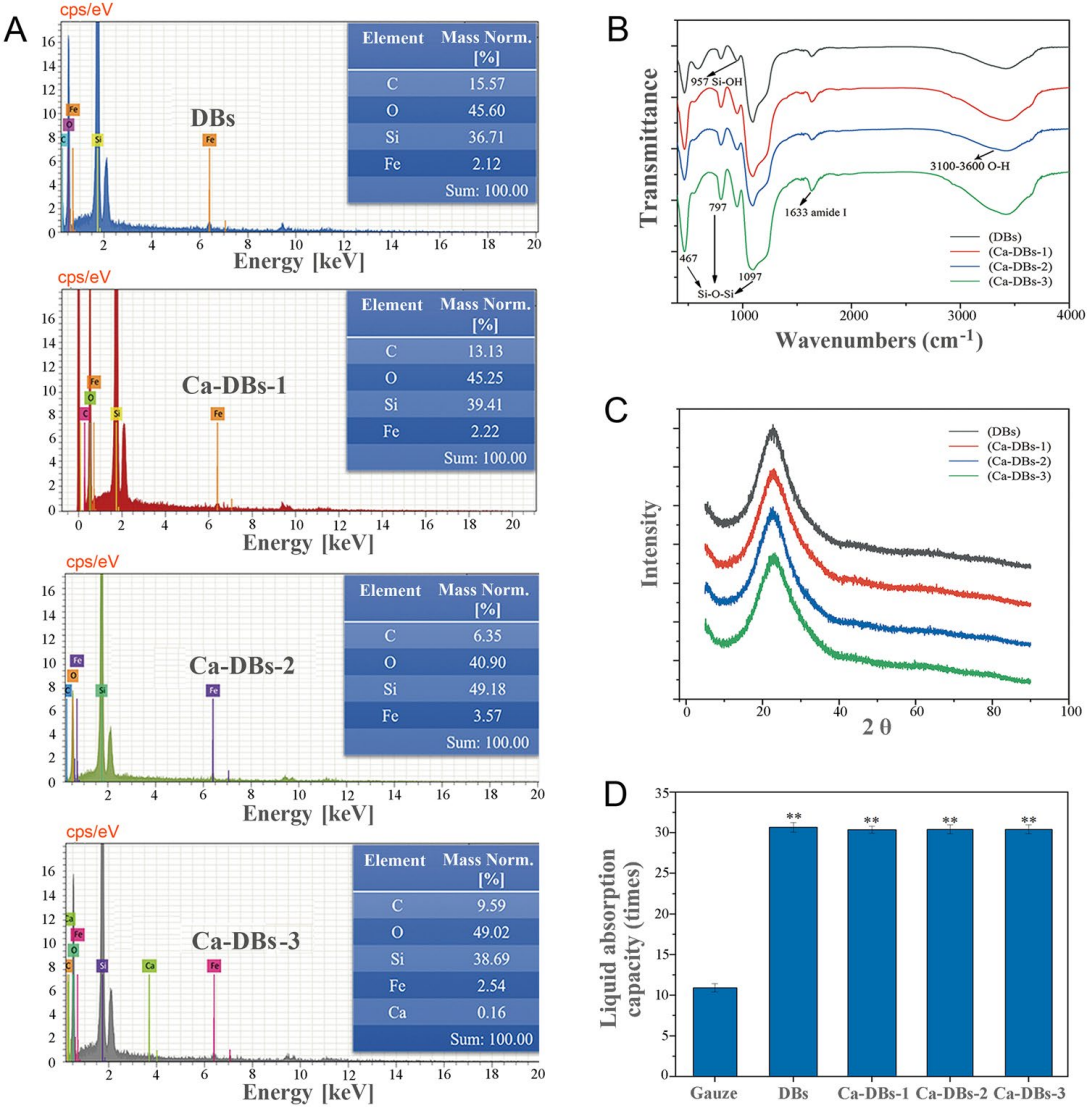
EDXS (**A**), FTIR (**B**), XRD analysis (**C**), and liquid absorption capacity (**D**) of DBs and Ca-DBs (Ca-DBs-1, Ca-DBs-2 and Ca-DBs-3). The data are expressed as mean ± SD (*n* = 3). ** represents statistical significance (*P* < 0.01)

The surface elements of DBs (control) and Ca-DBs (Ca-DBs-1, Ca-DBs-2, and Ca-DBs-3) were determined using EDXS. Elements O, Si, C, and Fe were contained in DBs and Ca-DBs (Fig. 2A). Ca^2+^ was detected only in Ca-DBs-3, indicating that 6.7 mmol L^−1^ CaCl_2_ affected the biomineralization of frustules, and 0.16% Ca^2+^ was biomineralized. The elemental content order was: O > Si > C > Fe > Ca.

According to the FTIR analysis, DBs and Ca-DBs (Ca-DBs-1, Ca-DBs-2, and Ca-DBs-3) exhibited similar band characteristics (Fig. 2B). The peaks at 467 cm^−1^, 797 cm ^−1^, and 1097 cm^−1^ were Si–O-Si. The peak at 957 cm^−1^ was Si–OH. The stretching vibration of the O–H produced broad and robust peaks between 3100 cm^−1^ and 3600 cm^−1^. The peak at 1633 cm^−1^ was associated with amide I (Zając et al. 2015).

XRD analysis revealed that DBs and Ca-DBs (Ca-DBs-1, Ca-DBs-2, and Ca-DBs-3) were composed of amorphous silica (Fig. 2C), implying that DBs and Ca-DBs could be considered safe biomedical materials (Monich et al. 2017). Figure 2D illustrates the capacity of DBs and Ca-DBs (Ca-DBs-1, Ca-DBs-2, and Ca-DBs-3) to adsorb SBF. The gauze was as a control. The liquid absorption capacity of DBs and Ca-DBs was three times that of gauze (*P* < 0.01). DBs and Ca-DBs had 30 times their dry weight in liquid absorption capacity. DBs and Ca-DBs showed no significant difference. SEM, TEM, FTIR, XRD, and the liquid absorption capacity of DBs and Ca-DBs (Ca-DBs-1, Ca-DBs-2, and Ca-DBs-3) were comparable. Due to the biomineralization of Ca^2+^ in Ca-DBs-3, DBs was used as a control for analyzing the coagulation and hemolysis of DBs and Ca-DBs-3.

### In vitro whole blood clotting time

The clotting time of DBs and Ca-DBs-3 was measured in vitro using whole blood (Supplementary Fig. S7A). Ca-DBs-3 had the shortest clotting time (163.80 ± 5.00 s). The hemostatic ability of Ca-DBs-3 is comparable to that of QuikClot^®^ zeolite. Ca-DBs-3 had a greater hemostatic effect than DBs (*P* < 0.05). Ca-DBs-3’s superior blood clotting ability benefits from many ways. First, the large micro-nano pores in Ca-DBs-3 have a high capacity for absorbing liquid, which allows for the rapid agglutination of coagulation factors and the acceleration of the coagulation cascade reaction (Na et al. 2011; Rhee et al. 2008). Second, Ca-DBs-3 contains large negatively charged polar silanol groups that interact actively with blood cells, promoting the formation of blood clots (Slowing et al. 2006). Third, the Ca^2+^ on the surface of Ca-DBs-3 acts as a coagulation factor IV, promoting blood coagulation further (Ratnoff and Potts 1954). Supplementary Fig. S7B depicts the SEM observation of blood clots. The blank control group’s erythrocytes had a typical disc shape, forming fibrin in the blood clot. Erythrocytes were adsorbed on the surface of the DBs with fibrin surrounded in the blood clot. Ca-DBs-3 adsorbed erythrocytes and fibrin networks were found in blood clots. The production of fibrin is an important step in blood coagulation. Fibrin acts as a net for blood cells, converting blood from a liquid to a solid state, thereby promoting blood clotting (Butenas and Mann 2002).

### Coagulation cascade activation pathway

The PT test primarily indicates the status of the exogenous coagulation system, whereas the aPTT test mainly shows the activity and function of endogenous coagulation factors (Kamal et al. 2007). For the PT, neither DBs nor Ca-DBs-3 exhibited significant differences from the control (Supplementary Fig. S7C). For the aPTT, both DBs and Ca-DBs-3 significantly reduced the reaction time (Supplementary Fig. S7D, *P* < 0.01), which was roughly 70%–80% shorter than the control, and Ca-DBs-3 was markedly faster than DBs (*P* < 0.01). The PT and the aPTT assays demonstrated that DBs and Ca-DBs-3 primarily promoted blood coagulation via the endogenous coagulation pathway. Negatively charged polar silanol groups on the surface of DBs and Ca-DBs-3 can activate coagulation factors (XII and XI) and bind to cofactors (prekallikrein and HWK-kininogen), increasing endogenous coagulation (Pourshahrestani et al. 2016). Additionally, the Ca^2+^ on the surface of Ca-DBs-3 was coagulation factor IV, enhancing the endogenous coagulation pathway (Chen et al. 2015).

### Viscoelasticity analysis of blood clots

TEG was used to assess the integrity of whole blood coagulation and guide the treatment of bleeding (Mohamed et al. 2017) (Supplementary Fig. S8). The R value for Ca-DBs-3 was 37% and 59% of the control and DBs, respectively (*P* < 0.05). The K value for Ca-DBs-3 was 27% and 68% of the control and DBs, respectively (*P* < 0.05). The angle for Ca-DBs-3 was 198% and 122% of the control and DBs, respectively (*P* < 0.05). No significant difference was found in MA between DBs and control, but Ca-DBs-3 was significantly greater than control by 18.4% (*P* < 0.05).

Both DBs and Ca-DBs-3 have the ability to stimulate blood coagulation start, expansion, and spread, with Ca-DBs-3 having a larger effect than DBs. The coagulation-promoting effect of Ca-DBs-3 is due to its microstructure. First, Ca-DBs-3 has a thick micro-nano layered pore structure and a high liquid absorption capacity, allowing it to rapidly agglutinate coagulation components and activate the coagulation cascade reaction (Na et al. 2011). Second, Ca-DBs-3 are silicon-based materials having a strong negative charge polarity silanol group, which aids the reaction at the blood cell interface, resulting in enriched blood cells and fibrin networks that form more tightly linked blood clots (Slowing et al. 2006). Third, the Ca^2+^ on the surface of Ca-DBs-3 is coagulation factor IV, which strengthens blood coagulation even more (Ratnoff and Potts 1954).

### Cytotoxicity and hemolysis rate of DBs and Ca-DBs-3

**Fig. 3:**
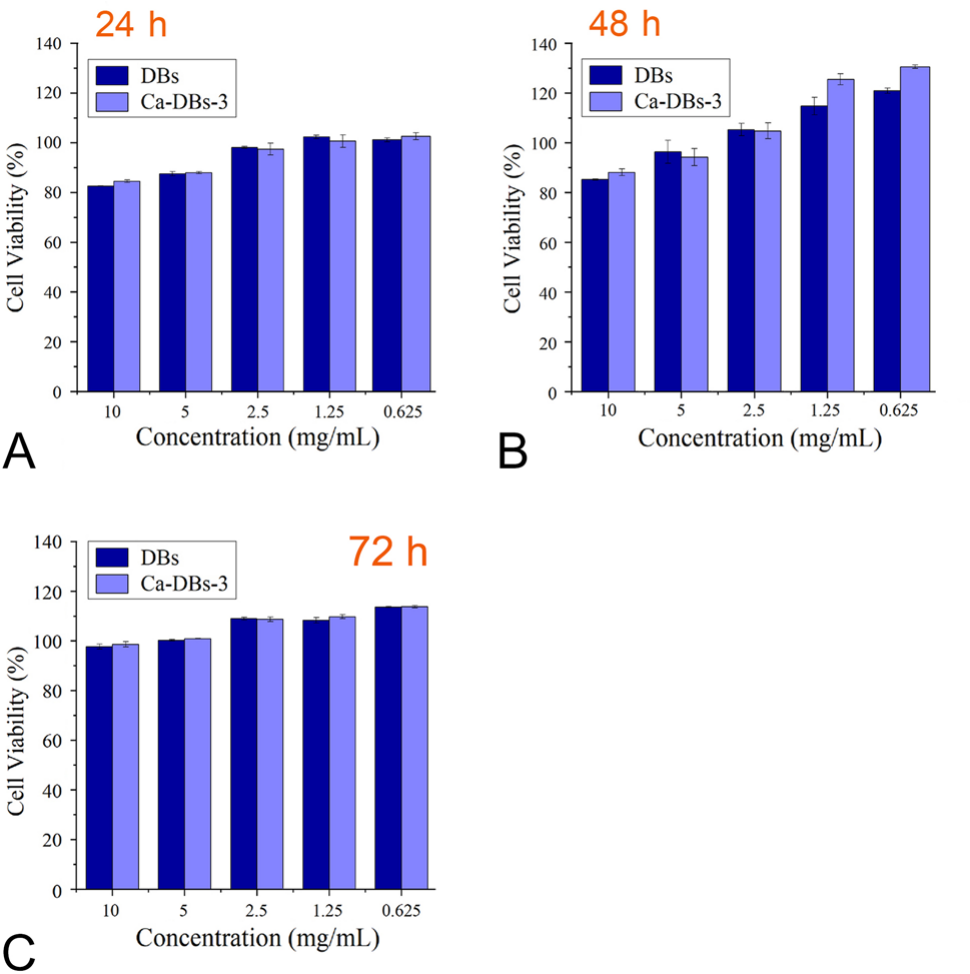
Cytotoxicity of DBs and Ca-DBs-3 on L929 cells. **A**, **B**, and **C** indicate the cell viability of L929 cells incubated with DBs and Ca-DBs-3 at different concentrations for 24, 48, and 72 h, respectively. The x-axis represents DBs and Ca-DBs-3’s concentration, and the y-axis represents L929 cell viability. The data are expressed as mean ± SD (*n* = 5)

DBs and Ca-DBs-3 were co-cultured in vitro with L929 cells to assess the cytotoxicity. The cytotoxicity increased as the sample concentration increased between 0.625 and 10 mg mL^−1^ (Fig. 3). After 24 h of culture, both DBs and Ca-DBs-3 exhibited mild cytotoxicity with over 80% of cells surviving. DBs and Ca-DBs-3 may be cytotoxic because negatively charged silanol groups interact with cells at the interface. The cell viability increased as the culture time increased. The cell viability remained greater than 100% at 72 h, indicating DBs and Ca-DBs-3 were cytocompatible.

**Fig. 4:**
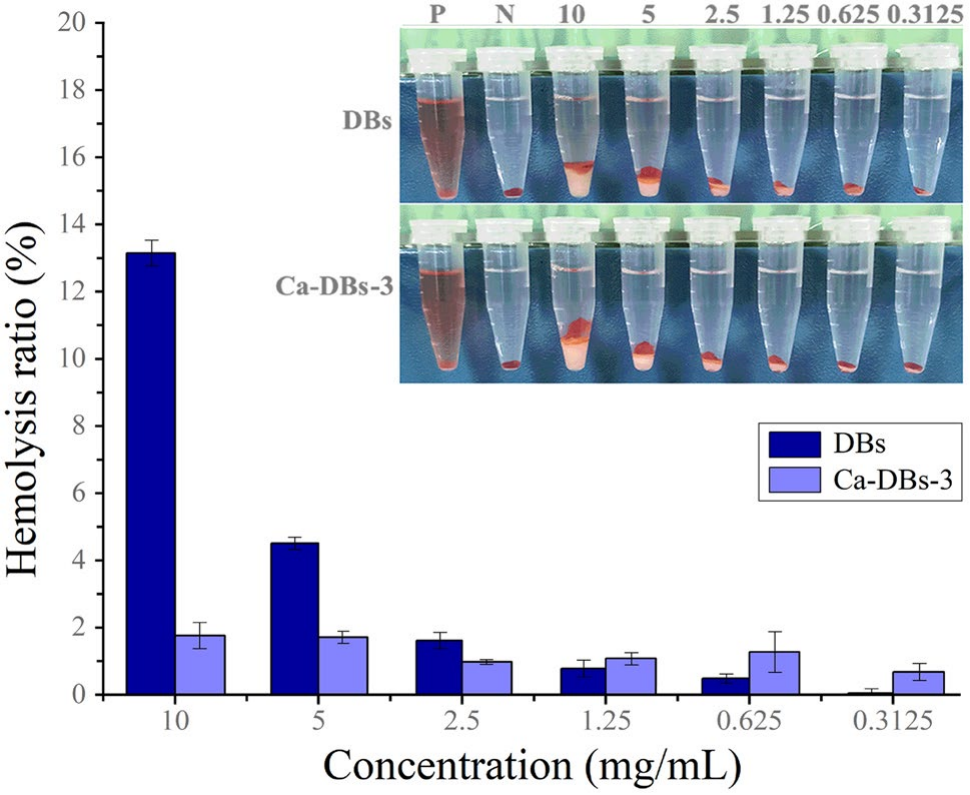
Hemolysis rate of DBs and Ca-DBs-3. The x-axis represents DBs and Ca-DBs-3’s concentration, and the y-axis represents the hemolysis ratio. P and N represent the positive and the negative control, respectively. Data are represented by mean ± SD (*n* = 3)

Hemolysis rate primarily reflects free hemoglobin concentration in plasma following complete contact with blood. Hemolysis criteria for biomaterials can be classified as non-hemolysis (hemolysis rate < 2%), mild hemolysis (hemolysis rate 2%-5%), or hemolysis (hemolysis rate > 5%) (Huang et al. 2016; Song et al. 2019). The hemolysis rate of DBs and Ca-DBs-3 is shown in Fig. 4. For DBs, the hemolysis rate increases as the DB’s concentration increases from 0.3125 to 10 mg mL^−1^. The hemolysis rate of DBs was less than 2% in the concentration range of 0.3125–2.5 mg mL^−1^, 4.51 ± 0.18% in 5 mg mL^−1^, and 13.15 ± 0.39% in 10 mg mL^−1^, respectively. The hemolysis rate for Ca-DBs-3 was always less than 2% in 0.3125–10 mg mL^−1^. Ca-DBs-3 had superior blood compatibility than DBs. The numerous negatively charged polar silanol groups on the surface of DBs and Ca-DBs-3 interact with erythrocytes and thus may lead to hemolysis. Ca-DBs-3’s hemolysis rate was lower than DBs, which may be related to its incorporation of Ca^2+^. Ca^2+^ may decrease Ca- Bs-3’s hemolysis rate by weakening its negative charge polarity.

### Hemostatic in vivo

**Fig. 5:**
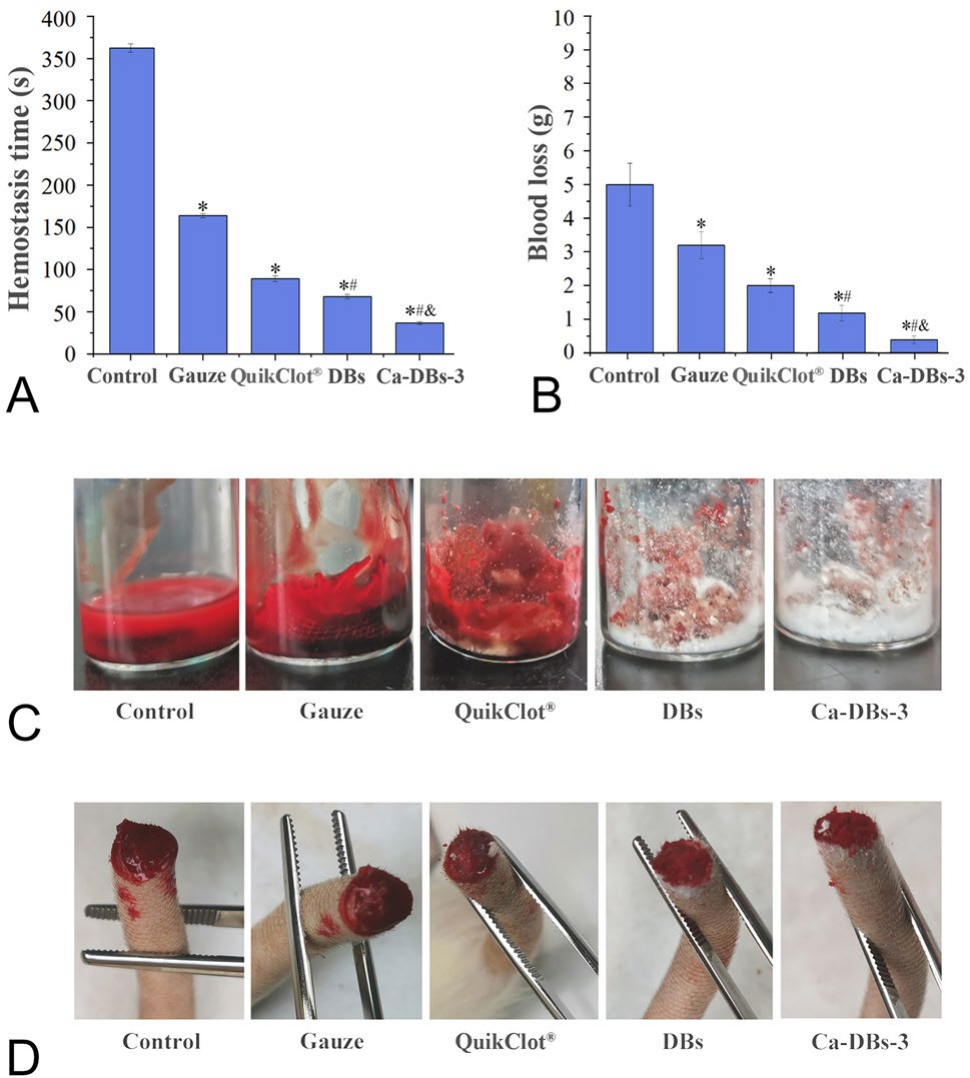
In vivo hemostatic time (**A**), blood loss (**B**–**C**), and the tail amputation hemostatic experiment (**D**) of DBs and Ca-DBs-3. Data are represented by mean ± SD (*n* = 5). *, # and & represent a significant difference from control, QuikClot^®^ zeolite and DBs (*P* < 0.05)

The hemostatic time and blood loss of DBs and Ca-DBs-3 in vivo were determined using a rat tail amputation model (Fig. 5). Ca-DBs-3 had the shortest hemostatic time (36.40 ± 2.52 s) and the least blood loss (0.39 ± 0.12 g). The in vivo hemostasis time of Ca-DBs-3 was 40.72% of QuikClot^®^ zeolite and 53.37% of DBs, and the blood loss of Ca-DBs-3 was 19.50% of QuikClot^®^ zeolite and 33.05% of DBs. The control group had gelatinous blood clots from the cross-section of the tail, whereas DBs and Ca-DBs-3 had no blood adsorption and stopped bleeding completely (Fig. 5D). The in vivo hemostatic test further supported the excellent hemostatic ability of Ca-DBs-3. Ca-DBs-3 possessed a stronger procoagulant effect than DBs. First, rich micro-nano pores endow Ca-DBs-3 with an exceptional capacity for liquid absorption, facilitating the rapid agglutination of coagulation factors and the initiation of the coagulation cascade (Na et al. 2011). Second, Ca-DBs-3 contains abundant negatively charged polar silanol groups that interact with blood cells, promoting the formation of blood clots (Slowing et al. 2006). Third, Ca^2+^ contained in Ca-DBs-3 is the coagulation factor IV, which aids in the coagulation reaction’s progression (Ratnoff and Potts 1954).

## Conclusions

In this work, CaCl_2_ was added to the diatom medium under 67.5 µmol m^−2^ s^−1^ (cool white, fluorescent lamps) to obtain Ca-DBs-3. Ca-DBs-3 was 40–50 μm in diameter and had hierarchical micro-nano pores: the first-order aperture was 1–1.5 μm; the second-order aperture was 200–250 nm; the third-order aperture was 50–100 nm. While the lower CaCl_2_ concentration had no apparent effect on the diatom’s carrying capacity, the higher CaCl_2_ concentration of 6.7 mmol L^−1^ inhibited the diatom’s carrying capacity significantly. The higher CaCl_2_ concentration influenced the frustules’ biomineralization, and Ca^2+^ was biomineralized in Ca-DBs-3 at a content of 0.16%. Ca-DBs-3 had the shortest hemostatic time (36.40 ± 2.52 s) and the least bleeding loss (0.39 ± 0.12 g) in the rat tail amputation model, which were 40.72% and 19.50% of QuikClot^®^ zeolite, respectively. Additionally, Ca-DBs-3 exhibited superior liquid absorption capacity, showed no apparent toxicity toward L929 cells (cell viability > 80%), and demonstrated good blood compatibility (hemolysis rate < 2%). In conclusion, Ca-DBs-3 has the potential to be developed into a rapid hemostatic material, laying the groundwork for developing the frustule-based hemostatic materials and the study of coagulation mechanisms.

## Materials and methods

### Conditions for diatom cultivation

The diatom *Coscinodiscus* sp. (CCAP 1013/11) was provided by the Ocean University of China’s Key Laboratory for Marine Genetics and Breeding. Diatoms were cultured at 21 °C, in 300 mL of high-pressure sterilized natural seawater (30 PPT) supplemented with F/2 (Guillard 1975), on a 12 h: 12 h light–dark cycle. Diatoms were cultured in a light incubator (GXZ-280B, Ningbo Jiangnan Instrument Factory, China). The type of lights were cool white, fluorescent lamps. Diatoms were acclimated in the exponential phase by serial transfers for 14 days prior to light and calcium chloride experiments. The first cultivation stage: set three light groups to 4.05, 40.5, and 67.5 µmol m^−2^ s^−1^ and the initial inoculation density of diatoms was identical. The diatoms were cultured for seven days. Each day, 1 mL diatom solution was collected concurrently to count cells. For the second stage of the cultivation, CaCl_2_ was added to seawater at three concentrations (1.675, 3.35, 6.7 mmol L^−1^) under 67.5 µmol m^−2^ s^−1^. The group lacking CaCl_2_ served as the control. Every 48 h, the number of diatoms was counted. Diatoms were harvested on the last day of cultivation.

### Frustule preparation and characterization

Diatoms were filtered on to a 500 Nitex mesh and then soaked in a solution containing 2 mol L^−1^ HCl and 30% H_2_O_2_ (V_HCl_:V_H2O2_ = 1:1) until a white precipitates (frustules) formed. The frustules were collected on 500 mesh Nitex mesh, washed three times with deionized water, and then vacuum dried for 24 h at 60 °C. Finally, frustules in light intensity group (DBs-4.05, DBs-40.5, and DBs-67.5) and CaCl_2_ concentration group (DBs, Ca-DBs-1, Ca-DBs-2, and Ca-DBs-3) were obtained.

Scanning electron microscopy (SEM, JSM-6010LA, JEOL, Japan) and transmission electron microscopy (TEM, H-9500, Hitachi, Japan) were used to examine the surface morphology and pore structure of frustules (DBs-4.05, DBs-40.5, and DBs-67.5, DBs, Ca-DBs-1, Ca-DBs-2, and Ca-DBs-3). The surface elements of frustules were determined using energy-dispersive X-ray spectroscopy (EDXS, SEM, JSM-6010LA, JEOL, Japan). The BET surface areas and pore diameters were determined by a Gas Sorption Analyzer (Autosorb-IQ, Konta, USA). Fourier transform infrared spectroscopy was used to analyze the chemical groups of frustules (FTIR, 5700, Nicolet, USA). A powder X-ray diffractometer was used to determine the crystallinity of frustules (XRD, SmartLab, Rigaku, Japan). The capacity of DBs, Ca-DBs-1, Ca-DBs-2, and Ca-DBs-3 to absorb liquid was determined in vitro using simulated body fluid (SBF) (Dai et al. 2010; Saxena et al. 2008). The liquid absorption capacity was determined using Eq. (1):

1 $$ {\text{Capacity for liquid adsorption }}\left( {\text{in times}} \right) = \left( {W_{wet} - W_{dry} } \right)/W_{dry} , $$

where *W*_*dry*_ and *W*_*wet*_ denote the weights of DBs and Ca-DBs before and after SBF soaked, respectively.

### In vitro evaluation of blood coagulation

The in vitro coagulation test was conducted following established methods (Behrens et al. 2014). Gauze, QuikClot^®^ zeolite, DBs, and Ca-DBs-3 (5 mg) were placed in 2 mL centrifuge tubes and incubated at 37 °C, respectively. Whole blood was extracted from the heart of a New Zealand white rabbit and injected into a tube containing 3.8% sodium citrate. The volume ratio of 3.8% sodium citrate to whole blood was 1: 9. Whole blood (1 mL) was added evenly to the centrifuge tubes containing the samples. Then 100 μL CaCl_2_ solution (0.2 mol L^−1^) was added, and the clotting time was recorded (i.e., the time for blood to clot completely). The tubes were rotated 180° every 10 s to determine whether the blood was clotted or not. The blank control was the whole blood without samples. Blood clots were washed three times with phosphate-buffered saline (PBS, pH 7.4) and fixed in 2.5% glutaraldehyde for 4 h. Finally, blood clots were dehydrated with gradient ethanol (30%, 50%, 70%, 90%, 100%) and observed by SEM after supercritical drying.

A semiautomatic coagulation analyzer was used to determine the prothrombin time (PT) and the activated partial thromboplastin time (aPTT) (TS6000, MD PACIFIC, China) (Wang et al. 2021a, b). Whole blood was extracted from the heart of a New Zealand white rabbit and injected into a tube containing 3.8% sodium citrate. Blood was centrifuged at 3000 rpm for 15 min to get the platelet plasma with a low platelet count (PPP). PPP (100 μL) was incubated at 37 °C for 180 s before being added to 100 μL PT reagent containing samples for the PT detection. At 37 °C for 180 s, aPTT (100 μL) reagent and PPP (100 μL) were co-incubated, and 100 μL CaCl_2_ (0.025 mol L^−1^) containing samples was added for the aPTT test. The control test was conducted without samples.

A thrombelastography analyzer was used to determine the viscoelasticity of blood clots (TEG 5000, Haemonetics Corporation, USA) (Wang et al. 2021a, b). Whole blood was extracted from the heart of a New Zealand white rabbit and injected into a tube containing 3.8% sodium citrate. The temperature of the TEG calibration was set to 37 °C. CaCl_2_ (20 μL, 0.1 mol L^−1^) was added into the TEG cup, followed by 340 μL blood samples (5 mg mL^−1^). The blood without samples was the blank control. The TEG test yielded four parameters: R (the time interval between the initial reaction and the formation of a measurable clot), K (the time interval between R and the construction of a clot with a specified hardness), angle (the rate of the clot formation), and MA (the maximum amplitude of the shear elasticity of the clot).

### In vitro biocompatibility evaluation

L929 cells viability was determined using CCK8 (Gao et al. 2016). L929 cells were cultured at 37 °C in 5% CO_2_ and seeded into 96-well plates (1 × 10^4^ cells well^−1^). The initial medium was replaced with the medium containing DBs and Ca-DBs (10, 5, 2.5, 1.25, 0.625 mg mL^−1^) after 12 h of cultivation. Cells cultured in the absence of samples (DBs and Ca-DBs) served as the control. The time intervals (24, 48, 72 h) were established for incubation. Following the completion of each culture, the wells were filled with the CCK8 solution (10 μL well^−1^) and incubated at 37 °C for 4 h. A micro-flat panel reader was used to determine the optical density of the supernatant at 450 nm (Sunrise, TECAN, Switzerland). The percentage of the surviving cells was calculated using Eq. (2):

2 $$ {\text{Cell survival rate}}\left( \% \right) = OD_{sample} /OD_{control} \times \, 100, $$

The hemolysis rate was tested according established methods (Zhao et al. 2011). DBs and Ca-DBs with different concentrations (10, 5, 2.5, 1.25, 0.625, 0.3125 mg mL^−1^) were prepared using saline in 1.5 mL centrifuge tubes and preheated at 37 °C. Whole blood was drawn from the heart of a New Zealand white rabbit and placed into a tube containing heparin sodium (heparin sodium: whole blood = 1:9). After that, the blood was diluted with saline (saline: blood = 5:4). Diluted blood (20 μL) was added to a suspension of 1 mL samples and thoroughly mixed. The mixture was then incubated at 37 °C for 1 h in a water bath. Following incubation, the tubes were centrifuged at 2000 rpm for 5 min, and optical density (545 nm) in the supernatant was measured by a micro flat-panel reader (Synergy HT, BioTek, USA). The positive and negative control were distilled water and saline, respectively. The hemolysis rate (%) was calculated using Eq. (3):

3 $$ {\text{Hemolysis rate}}\left( \% \right) = {{\left( {OD_s - OD_n } \right)} / {\left( {OD_p - OD_n } \right)}} \times 100, $$

where, *OD*_*s*_, *OD*_*p*_, and *OD*_*n*_ were the optical density of the supernatant of groups in samples, distilled water, and saline, respectively.

### In vivo hemostasis assay

Sprague Dawley rats (SD, 200–250 g, seven weeks old) were used for the tail amputation hemostasis experiment (Wang et al. 2019). Rats were anesthetized using a 10% chloral hydrate solution (0.005 mL g^−1^). The tail was cut (50% of the length), and 100 mg of the target material (gauze, QuikClot^®^ zeolite, DBs and Ca-DBs-3) was immediately applied to the wound. The time of hemostasis and the amount of blood loss were recorded.

### Statistical analysis

Data were expressed as mean ± standard deviation. One-way ANOVA was performed using Microsoft Excel 2019 MSO (16.0.14228.20216) 64 bits. The “*n*” in the legends represent the number of repetitions of the data. *P* < 0.05 represents a significant difference in the data.

## Supplementary Information

Below is the link to the electronic supplementary material.

Supplementary file1 (DOCX 11158 KB)

## Data Availability

The data supporting the findings of this study are available from the corresponding author upon reasonable request.
