# Supplementary material for: An improved blood hemorrhaging treatment using diatoms frustules, by alternating Ca and light levels in cultures
Source: Mar Life Sci Technol. 2023 Aug 18;5(3):316–25. doi: 10.1007/s42995-023-00180-3 (PMC10449749; doi:10.1007/s42995-023-00180-3)
Supplement: Supplementary file 1 — Supplementary file1 (DOCX 11158 KB) [file 42995_2023_180_MOESM1_ESM.docx]

**Title**

An improved blood hemorrhaging treatment using diatoms frustules, by alternating Ca and light levels in cultures

**Authors­**

Qinfeng Li^1^ · Zheng He^1^ · Hussein. E. Rozan^1,3^ · Chao Feng^1^ · Xiaojie Cheng^1^ · Xiguang Chen^1,2^

Corresponding author Xiaojie Cheng E-mail: xjcheng@ouc.edu.cn

Corresponding author Xiguang Chen E-mail: [xgchen@ouc.edu.cn](mailto:xgchen@ouc.edu.cn)

1. College of Marine Life Science, Sanya Oceanographic Institution, Ocean University of China, Qingdao 266003, China

2. Laoshan Laboratory, Qingdao 266237, China

3. Department of Biochemistry, Faculty of Agriculture, Al-Azhar University, Cairo, Egypt


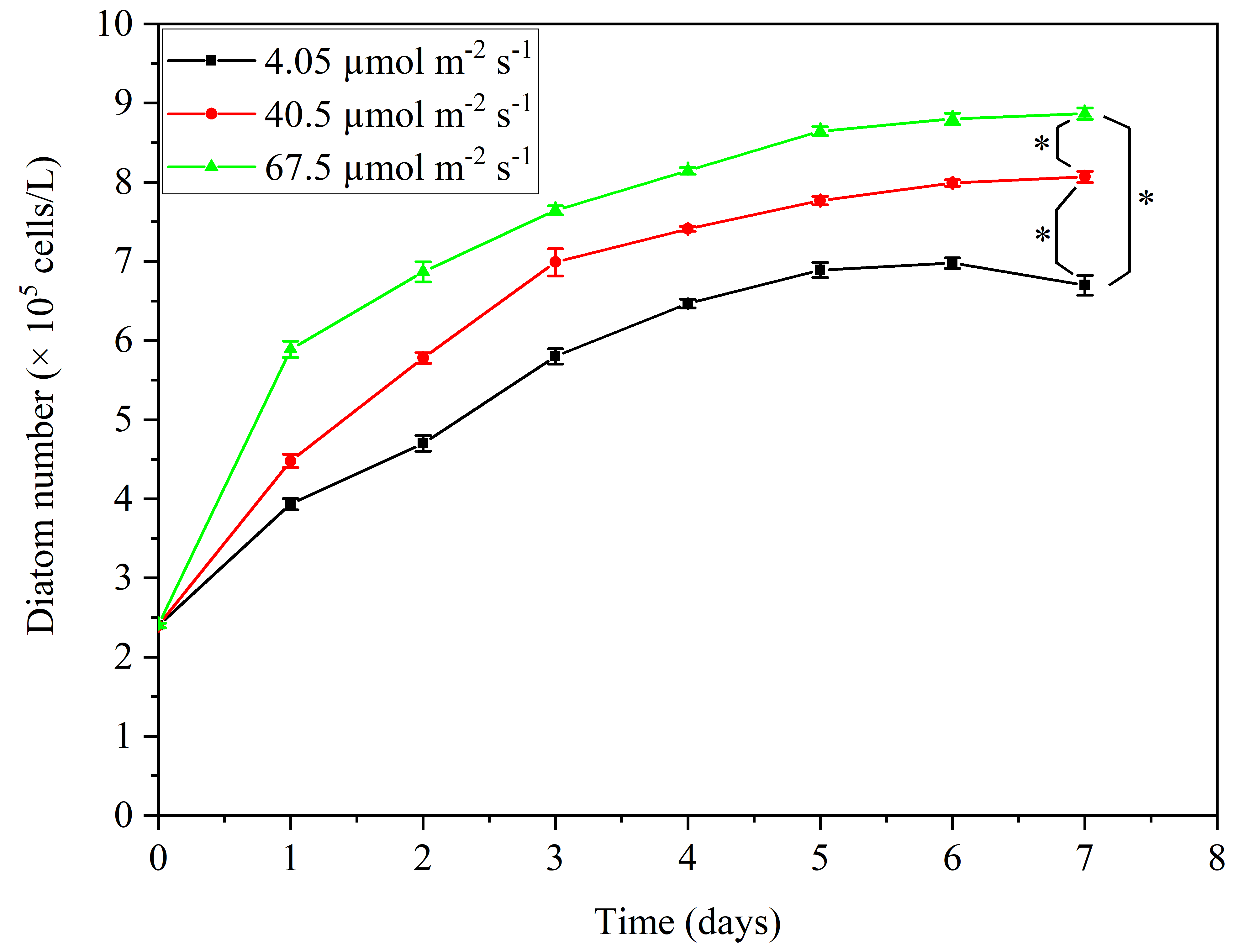


Supplementary Fig. S1 Growth curves of diatoms under 4.05, 40.5, and 67.5 µmol m^-2^ s^-1^ (cool white fluorescent lamps), respectively. The data are represented as mean ± SD (*n* = 3). * represents significant data differences between groups (*P < 0.05*).


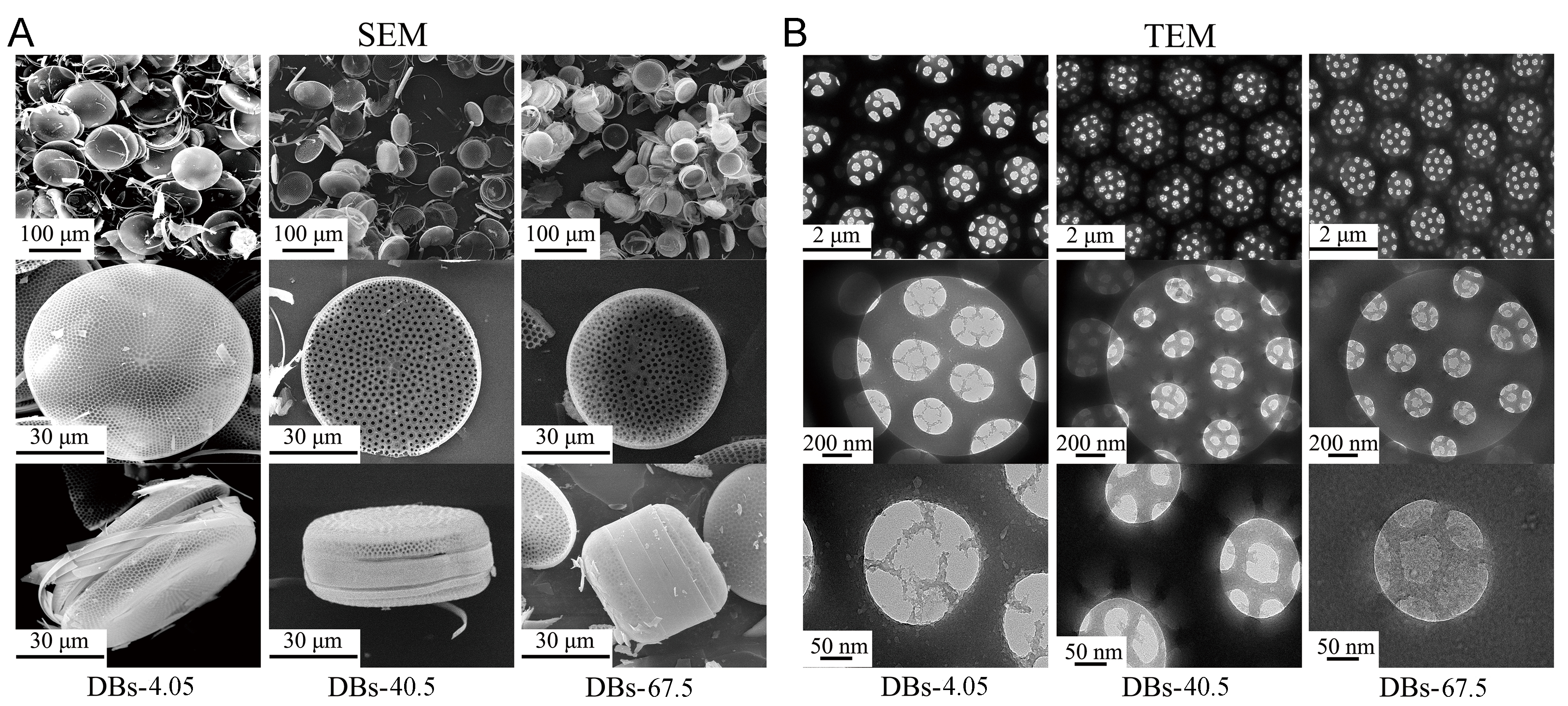


Supplementary Fig. S2 SEM and TEM observation of DBs-4.05, DBs-40.5, and DBs-67.5. DBs-4.05, DBs-40.5, and DBs-67.5 had diameters of 90-100 μm, 50-60 μm, and 40-50 μm, respectively. DBs-4.05, DBs-40.5, and DBs-67.5 have three-stage pore structures: primary pores 1-1.5 μm, secondary pores 200-250 nm, and tertiary pores 50-100 nm. The software calculated the data (Nano measurer 1.2, *n = 20*).


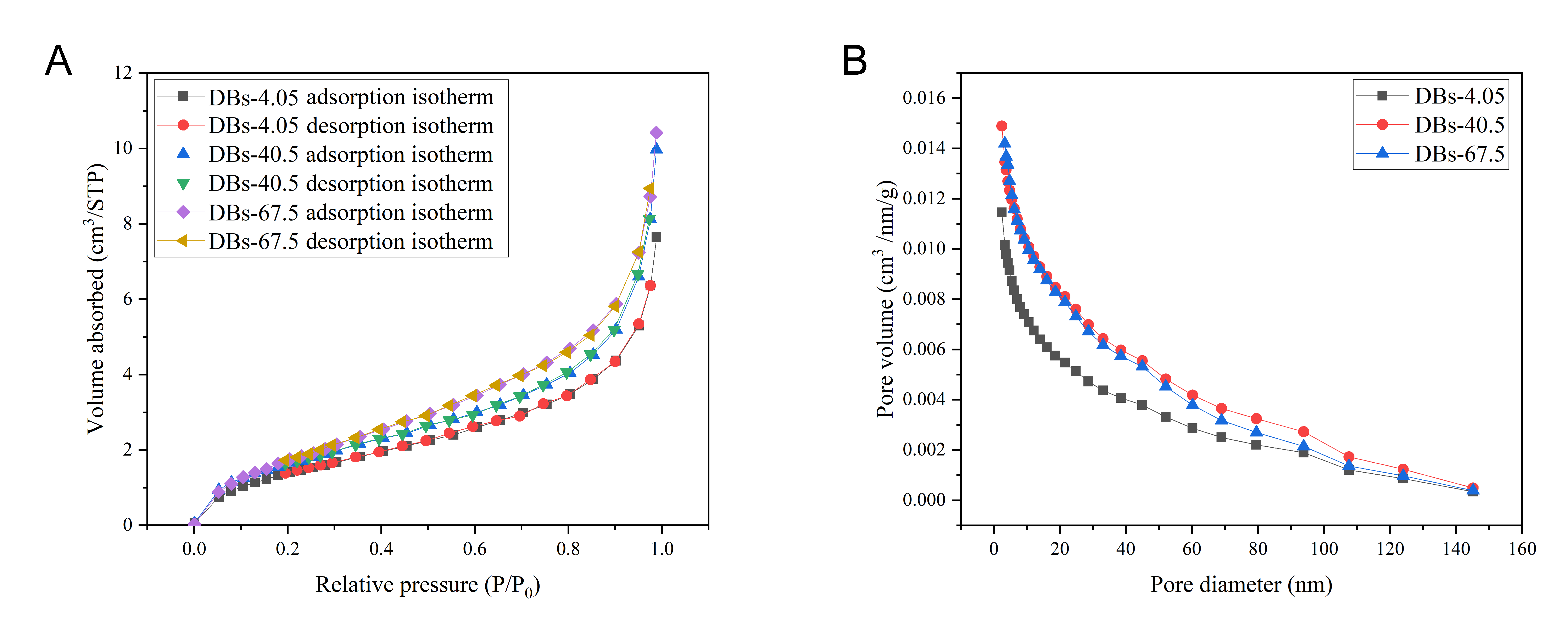


Supplementary Fig. S3 N_2_ adsorption-desorption isotherm (A) and pore size distribution of frustules (B)

Supplementary Table S1 Physical characteristics of DBs-4.05, DBs-40.5 and DBs-67.5.

| Sample | Size (μm) | BET  (m^2^ g^-1^) | Average pore diameter (nm) | BJH pore diameter (nm) | Total pore volume (cm^3^ g^-1^) |
| --- | --- | --- | --- | --- | --- |
| DBs-4.05 | 90-100 | 5.6416 | 8.3803 | 2.41 | 0.011819 |
| DBs-40.5 | 50-60 | 6.4358 | 9.5813 | 2.41 | 0.015416 |
| DBs-67.5 | 40-50 | 7.2578 | 8.8827 | 2.39 | 0.016117 |


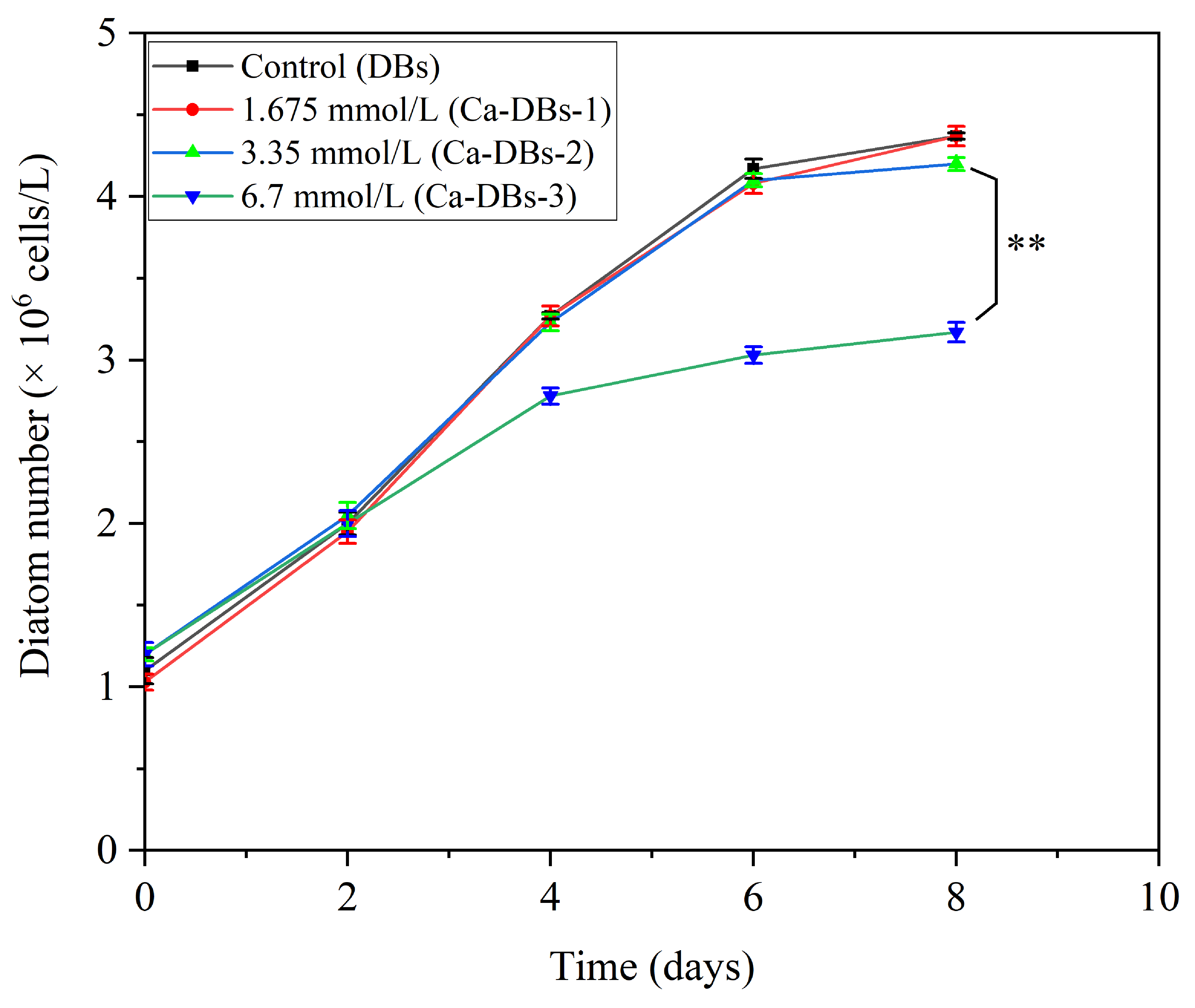


Supplementary Fig. S4 Diatom growth curves under 67.5 µmol m^-2^ s^-1^ (cool white, fluorescent lamps). CaCl_2_ concentrations of 1.675, 3.35, and 6.7 mmol/L were added to the F/2 medium, respectively. The group lacking CaCl_2_ served as the control. Data are represented by mean ± SD (*n* = 3). ** indicates a statistically significant difference between groups (*P* < 0.01).


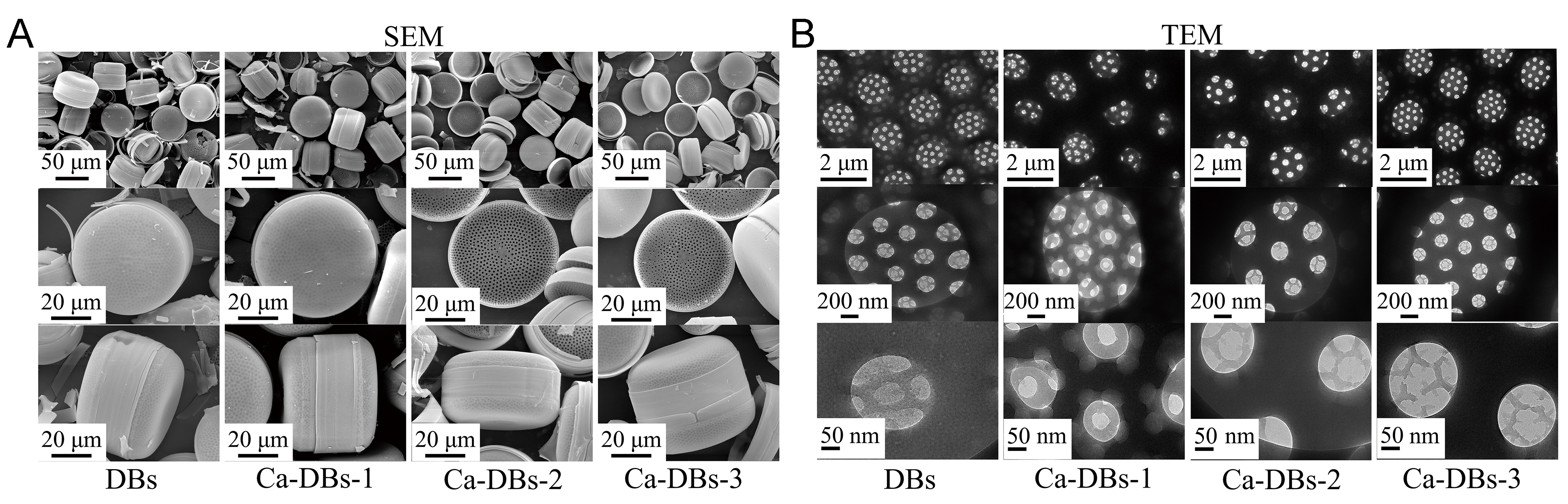


Supplementary Fig. S5 DBs and Ca-DBs (Ca-DBs-1, Ca-DBs-2, and -DBs-3) morphology observation using SEM and TEM. DBs and Ca-DBs had a diameter of 40-50 μm, with three-stage pore structures: primary pores 1-1.5 μm, secondary pores 200-250 nm, and tertiary pores 50-100 nm. The data were analyzed using a software program (Nano measurer 1.2, *n = 20*).


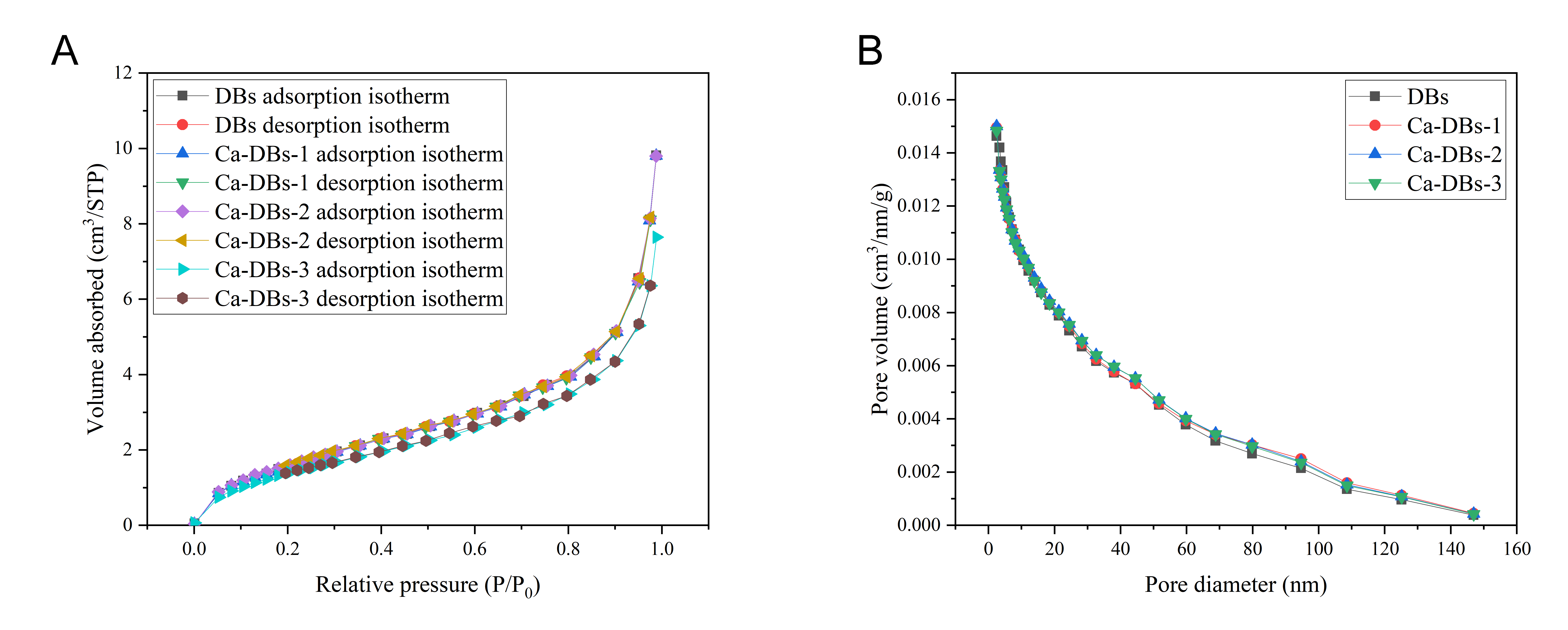


Supplementary Fig. S6 N_2_ adsorption-desorption isotherm (A) and pore size distribution of frustules (B)

Supplementary Table S2 Physical characteristics of DBs, Ca-DBs-1, Ca-DBs-2 and Ca-DBs-3.

| Sample | Size (μm) | BET  (m^2^ g^-1^) | Average pore diameter (nm) | BJH pore diameter (nm) | Total pore volume (cm^3^ g^-1^) |
| --- | --- | --- | --- | --- | --- |
| DBs | 40-50 | 6.4874 | 9.3559 | 2.43 | 0.015174 |
| Ca-DBs-1 | 40-50 | 6.7448 | 8.9891 | 2.43 | 0.015157 |
| Ca-DBs-2 | 40-50 | 6.8357 | 8.8652 | 2.41 | 0.015150 |
| Ca-DBs-3 | 40-50 | 6.4697 | 8.4802 | 2.41 | 0.015241 |


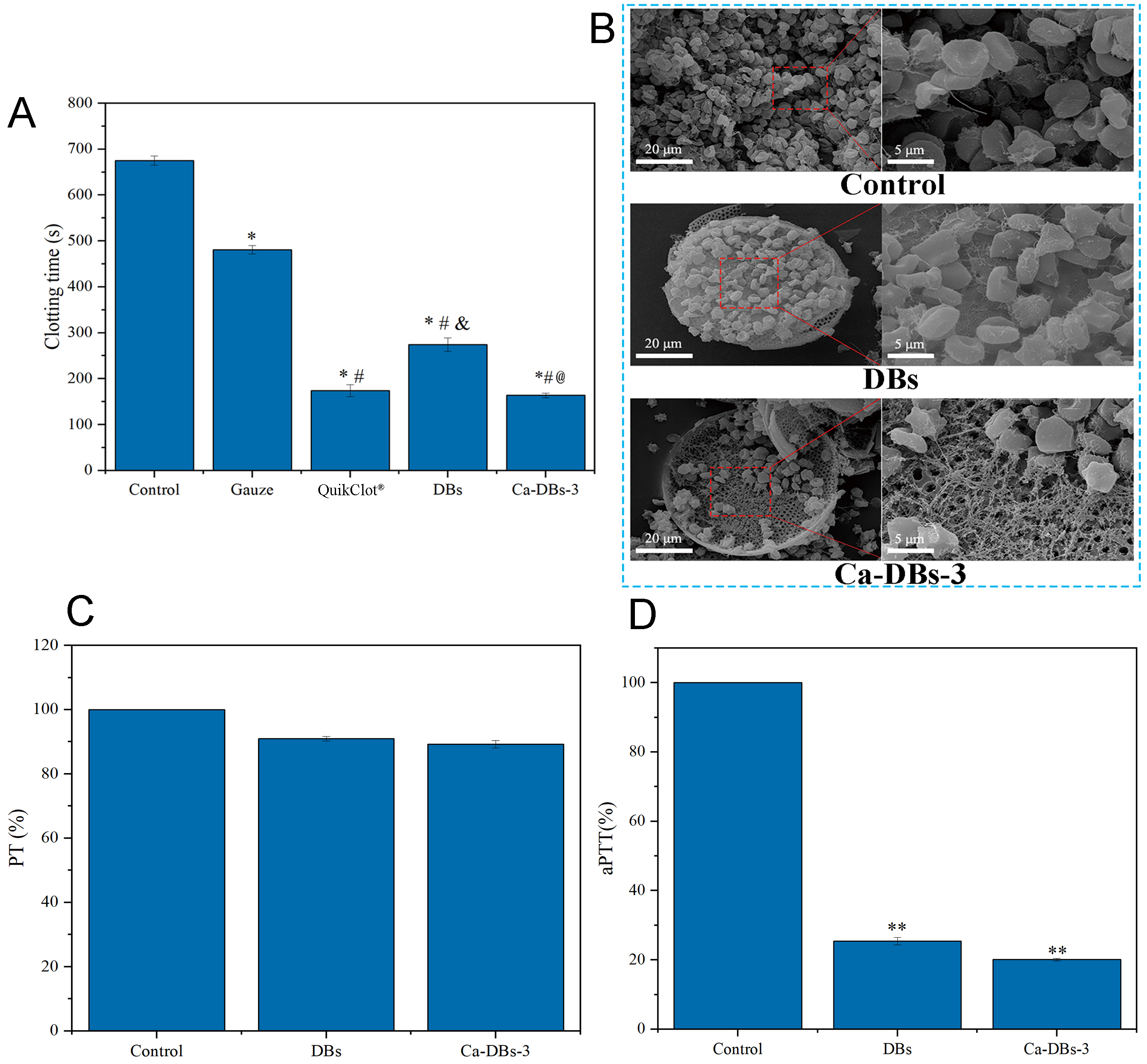


Supplementary Fig. S7 *In vitro* whole blood clotting time (A), blood clots (B), PT (C), and aPTT (D) of DBs and Ca-DBs-3. The data are expressed as mean ± SD (*n* = 5). *, #, & and @ represent the significant difference from control, gauze, QuikClot^®^ zeolite and DBs respectively (*P* < 0.05). ** *P* < 0.01.


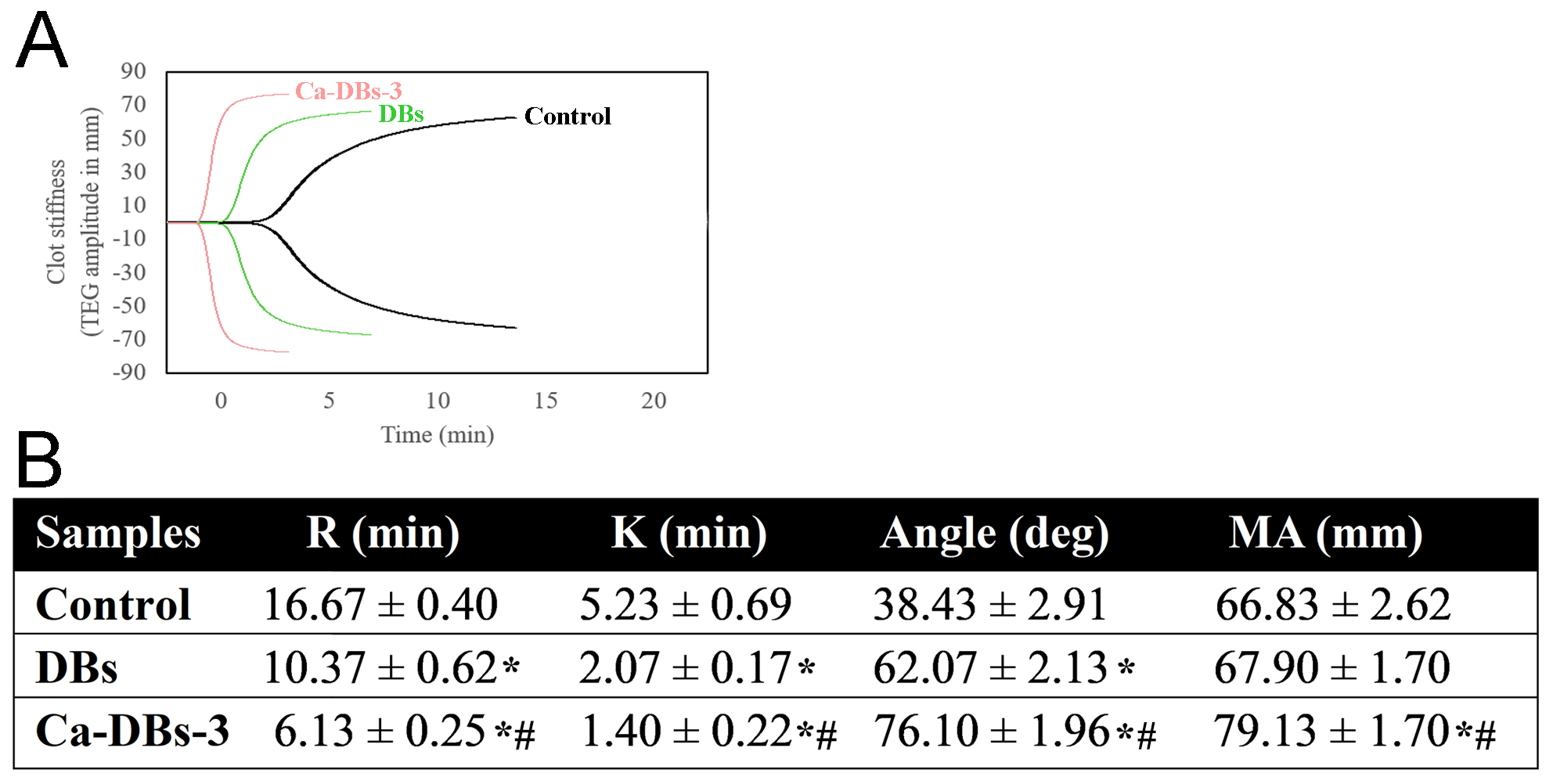


Supplementary Fig. S8 TEG curves (A) and parameters (B) of DBs and Ca-DBs-3. Data are represented by mean ± SD (*n* = 3). * and # represent significant differences from control and DBs (*P* < 0.05).
